# *Mycoplasma penetrans* urethritis in men. A case–control study

**DOI:** 10.3389/fmicb.2025.1565685

**Published:** 2025-03-19

**Authors:** Luis Piñeiro, Pedro Idigoras, Ayla Manzanal, Iñigo Ansa, Diego Vicente

**Affiliations:** Department of Microbiology, Donostia University Hospital, Biogipuzkoa Health Research Institute, OSI Donostialdea, University of the Basque Country (UPV/EHU), San Sebastián, Spain

**Keywords:** *Mycoplasma penetrans*, emerging pathogen, sexually transmitted infection, idiopathic urethritis, men

## Abstract

**Introduction:**

A microbiological diagnosis is not reached in many urethritis cases, the proportion varying with the diagnostic methods and targets available. *Mycoplasma penetrans* is an emerging pathogen, recently described as a possible aetiological agent in urethritis, especially in men who have sex with men (MSM) and persons living with HIV.

**Methods:**

Between June 2021 and June 2024, urethral samples from men were analysed for the presence of *M. penetrans* using an in-house real-time PCR, and for other sexually transmitted infections with standard techniques (gram stain, culture, PCR, and serology). Three groups were studied, one comprising 55 consecutive cases of urethritis in which the infectious aetiology had not previously been identified, and two randomly obtained control groups: 102 patients with microbiologically-identified urethritis, and 91 patients with no manifestations of urethritis and no pathogen detected.

**Results and discussion:**

*M. penetrans* DNA was detected in 7/55 (12.7%) of the idiopathic urethritis cases, but not in any of the controls (*p* < 0.001). None of the *M. penetrans*-positive patients had HIV infection and six were MSM. The results from this study indicate an association between infection by *M. penetrans* and urethritis in men. Therefore, the use of techniques for detecting *M. penetrans* could help bridge the diagnostic gap in idiopathic urethritis.

## Introduction

Exudative sexually transmitted infections (STIs), urethritis and cervicitis, are a global public health concern, and their incidence has increased over the last decade (Rowley et al., 2015; Workowski et al., 2021; ECDC, 2024; CNE, 2024). The most common aetiological agents of these infections are *Chlamydia trachomatis*, *Neisseria gonorrhoeae* and *Mycoplasma genitalium* but some cases involve other pathogens such as *Ureaplasma urealyticum*, *Haemophilus influenzae*, herpes simplex virus, adenovirus or *Trichomonas vaginalis*. The detection and specific identification of these microorganisms is crucial not only for appropriately treating the diseases they cause but also for stopping their spread to sexual partners of the infected individuals. Delayed and imprecise diagnosis increase the likelihood of both transmission to sexual partners and long-term complications (Workowski et al., 2021; Piñeiro et al., 2019).

Nowadays, the great variety of methods available for microbiological diagnosis makes it possible to reach an aetiological diagnosis in many cases, though the reported percentage varies greatly depending on the diagnostic capacity of the laboratory (50–80%) (Workowski et al., 2021). Nevertheless, there are still cases with clear signs or symptoms of urethritis in which, after ruling out noninfectious causes, an aetiological diagnosis is not obtained (idiopathic urethritis), and these have to be empirically treated. Among the microorganisms potentially involved, *Mycoplasma penetrans* has recently been associated with 13% of cases of urethritis in men who have sex with men (MSM), a finding that could expand the diagnostic landscape for STIs (Srinivasan et al., 2021).

The objective of this study was to investigate the potential association between *M. penetrans* infection and cases of idiopathic urethritis in men, either as a primary pathogen or in collaboration with other microorganisms, and to explore related clinical and epidemiological characteristics.

## Materials and methods

### Study setting

An observational case–control study was carried out between June 2021 and June 2024, with data gathered retrospectively up to June 2022, and thereafter, prospectively. The presence of *M. penetrans* DNA was assessed in urethral samples from patients who attended the Microbiology Service at Donostia University Hospital (Gipuzkoa, Spain; catchment population of around 650,000 people). Data were gathered on clinical and epidemiological characteristics (age, origin, sexual behavior, and symptoms, among others), and samples were collected (urethral, first-catch urine, serum and extragenital samples, based on clinical exploration and sexual behavior).

Patients were classified into one of three study groups: (1) all consecutive cases with idiopathic urethritis (with symptoms and/or signs); (2) controls with urethritis and in whom a well-established causal agent (*C. trachomatis, N. gonorrhoeae, M. genitalium*, etc.) had been identified, to assess possible coinfections with *M. penetrans*; and (3) asymptomatic controls in whom no pathogen had been detected, either post-treatment or in contact tracing studies, to rule out the possibility of *M. penetrans* being a member of the commensal microbiota of the urethra. Patients from groups 2 and 3 were randomly selected whenever a group 1 case was identified (at a ratio of ~2:1). With the expected prevalences for each group (10% for group 1, and 1% for groups 2 and 3) (Srinivasan et al., 2021), it was estimated that a minimum of 50 patients were needed in each group to obtain statistically significant results.

Patients with *M. penetrans* infection were treated with a single-dose regimen of 1 g of azithromycin and invited for a test of cure after 1–3 months. Contact tracing study and any other STIs diagnosed were managed following current guidelines (Workowski et al., 2021).

### Diagnostic techniques

A Gram stain was done on the urethral and first-catch urine samples immediately after collection during the patient’s visit, and subsequently, flow cytometry was performed using the same urine sample (XN-350 SISMEX, Spain). The information obtained about the microorganisms observed and/or the presence of leucocytes (white blood cell count and the polymorphonuclear leucocyte percentage), were used to guide the treatment of each patient. The urethral sample was cultured on blood agar (Columbia agar +5% sheep blood, bioMérieux, Marcy-l’Etoile, France), and two types of chocolate agar (GC II Agar with BD IsoVitaleX™, Becton Dickinson, USA; and Chocolate agar + PolyViteX™ VCAT3 agar, bioMérieux, Marcy-l’Etoile, France), and incubated for 48 h at 35°C in an atmosphere of 5% CO_2_.

For the molecular detection of STI microorganisms, the urethral swab was immersed in a viral transport medium and mixed with 1 mL of the urine sample. Nucleic acids were extracted in a STARlet (Hamilton, Houston, USA) system, and analysed using a syndromic testing kit (Allplex™ STI Essential Assay, Seegene, Seoul, South Korea), which detects DNA from *C. trachomatis*, *N. gonorrhoeae*, *M. genitalium*, *U. urealyticum* and *T. vaginalis.* In cases of urethritis that yielded negative results for all these pathogens, additional assays were performed, using a VIASURE multiplex Herpes virus 1, Herpes virus 2 and *Treponema pallidum* Real Time PCR Detection Kit (Certest Biotec, Zaragoza, Spain) and an in-house PCR for the detection of adenovirus that amplifies a fragment of the F gene (Xu et al., 2000).

*M. penetrans* DNA was detected using an in-house RT-PCR using the primers and probe previously described by Srinivasan et al. (2021). The following reaction mixture, optimized for our laboratory setting, was used: 15 μL of PCR mix [7.4 μL dH_2_O, 1.6 μL Cl_2_Mg 25 mM, 1 μL of the primer 56-F at 5 μM, 1 μL of the primer 184-R at 5 μM, 2 μL of probe MP-FAM at 2 μM and 2 μL of DNA Master Mix (Roche, Mannheim, Germany)] and 5 μL of DNA sample. RNAse-free water was used as a negative control and *M. penetrans* DNA (DSM 22633, DSMZ, Germany) as a positive control. The temperature conditions were 95°C for 10 min, followed by 50 cycles of 95°C for 15 s and 60°C for 1 min. The assay was performed in a LightCycler 2.0 system (Roche Diagnostics, Mannheim, Germany).

### Statistical analysis

Statistical analysis was performed using IBM SPSS statistics software (version 23, IBM, Chicago, IL, USA). Probabilities or proportions were compared between groups using the Odds ratio or Chi-square test (applying the Yates correction when necessary), respectively. Continuous distribution was analyzed with the Student’s *t* test. Values of *α* ≤ 0.05 were considered statistically significant.

## Results

A total of 248 urethral samples were analysed from men (median age: 34 years, range: 16–66 years) (Table 1 and Supplementary Table S1). Of these, 119 (48%) samples were from MSM and 129 (52%) from men who have sex with women (MSW). Overall, 188 samples were from Spanish-born men and 60 were from men born elsewhere (24.1% in other European countries, and 51.7, 20.7 and 3.4% in South and Central America, Africa and Asia respectively). The group distribution was as follows: 55 patients in group 1, 102 in group 2 and 91 in group 3. The 55 cases with idiopathic urethritis (group 1) represented 4.6% of all cases of urethritis documented during the study period.

**Table 1:** Epidemiological and clinical data of the population studied, by study group and *Mycoplasma penetrans* status.

	Group 1^#^		Group 2		Group 3		Total
	MP+	MP-	MP+	MP-	MP+	MP-	
	*N* = 7 (%)	*N* = 48 (%)	*N* = 0 (%)	*N* = 102 (%)	*N* = 0 (%)	*N* = 91 (%)	*N* = 248 (%)
Sexual behavior							
MSM	6 (85.7)	31 (64.6)	0 (0.0)	33 (32.4)	0 (0.0)	49 (53.8)	119 (48.0)
MSW	1 (14.3)	17 (35.4)	0 (0.0)	69 (67.6)	0 (0.0)	42 (46.2)	129 (52.0)
Age (years)							
<30	1 (14.3)	18 (37.5)	0 (0.0)	45 (44.1)	0 (0.0)	35 (38.5)	99 (39.9)
≥30	6 (85.7)	30 (62.5)	0 (0.0)	57 (55.9)	0 (0.0)	56 (61.5)	149 (60.1)
Origin							
Spanish born	5 (71.4)	32 (66.7)	0 (0.0)	77 (75.5)	0 (0.0)	74 (81.3)	188 (75.8)
Foreign born	2 (28.6)	16 (33.3)	0 (0.0)	25 (24.5)	0 (0.0)	17 (18.7)	60 (24.2)
Gram stain (PMNs/HPF)*							
<5	1 (14.3)	6 (12.5)	0 (0.0)	12 (11.8)	0 (0.0)	91 (100)	110 (44.4)
≥5	6 (85.7)	42 (87.5)	0 (0.0)	90 (88.2)	0 (0.0)	0 (0.0)	138 (55.6)
History of STI							
No	2 (28.6)	21 (43.8)	0 (0.0)	65 (63.7)	0 (0.0)	27 (29,7)	115 (46.4)
*Chlamydia trachomatis*	2 (28.6)	10 (20.8)	0 (0.0)	16 (15.7)	0 (0.0)	29 (31.9)	57 (22.9)
*Neisseria gonorrhoeae*	3 (42.9)	16 (33.3)	0 (0.0)	17 (16.7)	0 (0.0)	20 (21.9)	56 (22.6)
*Mycoplasma genitalium*	3 (42.9)	7 (14.6)	0 (0.0)	5 (4.9)	0 (0.0)	13 (14.3)	28 (11.3)
HIV-positive	0 (0.0)	3 (6.3)	0 (0.0)	3 (2.9)	0 (0.0)	7 (7.7)	13 (5.2)
*Treponema pallidum*	2 (28.6)	4 (8.3)	0 (0.0)	3 (2.9)	0 (0.0)	14 (15.4)	20 (8.1)
Other**	1 (14.3)	1 (2.1)	0 (0.0)	2 (2.0)	0 (0.0)	5 (5.5)	9 (3.6)
Urethral pathogen detected							
*Chlamydia trachomatis*	0 (0.0)	0 (0.0)	0 (0.0)	55 (53.9)	0 (0.0)	0 (0.0)	55 (22,2)
*Neisseria gonorrhoeae*	0 (0.0)	0 (0.0)	0 (0.0)	31 (30.4)	0 (0.0)	0 (0.0)	31 (12,5)
*Mycoplasma genitalium*	0 (0.0)	0 (0.0)	0 (0.0)	8 (7.8)	0 (0.0)	0 (0.0)	8 (3,2)
*Ureaplasma urealyticum*	0 (0.0)	0 (0.0)	0 (0.0)	6 (5.9)	0 (0.0)	0 (0.0)	6 (2,4)
Adenovirus	0 (0.0)	0 (0.0)	0 (0.0)	4 (3.9)	0 (0.0)	0 (0.0)	4 (1,6)
Herpes simplex virus	0 (0.0)	0 (0.0)	0 (0.0)	3 (2.9)	0 (0.0)	0 (0.0)	3 (1,2)

* ≥5 PMNs/HPF in urethra and/or ≥ 20/ml leukocytes in urine.

**Other pathogens are listed in Supplementary Table S1.

^#No statistical differences were found using Chi-square or odds ratio tests.^

MSM, men who have sex with men; MSW, men who have sex with women; PMN, polymorphonuclear leukocyte; HPF, high-power field; STI, sexually transmitted infection; HIV, human immunodeficiency virus; MP+, *Mycoplasma penetrans*-positive; MP-, *Mycoplasma penetrans*-negative.

**Figure 1:**
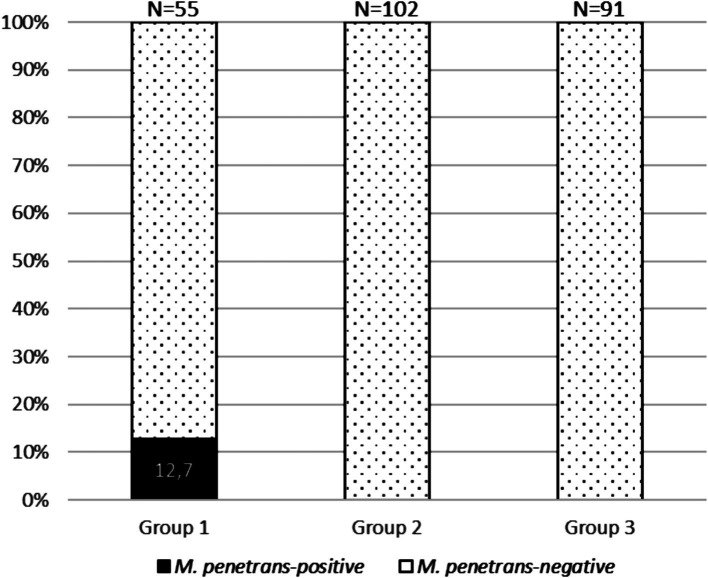
Stacked bar chart representing the prevalence of *Mycoplasma penetrans*-positive cases across study groups.

Across the 3 years of the study, *M. penetrans* was detected in 7 (12.7%, 95% CI 6.4–24.1) cases from group 1 and none of the controls (groups 2 or 3) (*p* < 0.001); 6/37 (16.2%) cases were in MSM and 1/18 (5.6%) in MSW (*p* = NS) (Figure 1 and Supplementary Figure S1). In the seven *M. penetrans*-positive cases of urethritis (Table 2), the patients had a median age of 38 years (vs 34 years, *p* = NS), five were Spanish, five had history of STI and none were living with HIV. As well as urethritis, one of the patients presented with three elevated dermic lesions on the balanopreputial groove, in which monkeypox virus was detected. Six patients were successfully treated initially with a single dose of 1 g of azithromycin; however, in one case, a second dose was required after reinfection through an untreated unknown sexual partner, and another patient required a second treatment with azithromycin using an extended regimen (500 mg as a single dose, followed by 250 mg/day for 4 days) due to relapse. In the seventh case, the infection resolved spontaneously, with no need for antimicrobial treatment. No epidemiological link was found between the cases. Contact tracing study was performed in three cases (one symptomatic and two asymptomatic), in which *M. penetrans* was not detected in neither urethral nor rectal samples.

**Table 2:** Epidemiological and clinical features of the seven *Mycoplasma penetrans*-positive men with urethritis.

Case	Date	Age (years)	Sexual behavior	Previous STIs	Symptoms	Treatment	Course
1	2 June 2021	30	MSM	MG	Dysuria and discharge	None	Resolution 5 days after consultation
2	19 Jan 2022	38	MSM	HSV-2	Dysuria and discharge	Azithromycin 1 g	Resolution after 15 days with persistent dysuria
3	11 Sept 2023	30	MSM	No	Dysuria and discharge	Azithromycin 1 g	Resolution
4	19 Dec 2023	54	MSM	NG	Dysuria	Azithromycin 1 g	Resolution
5	27 May 2024	43	MSM	CT, NG, MG, TP	Inguinal adenopathy and mpox penile lesions	Azithromycin 1 g	Resolution
6	30 May 2024	20	MSW	No	Dysuria and discharge	Azithromycin 1 g	Mild recurrence after 15 days with discharge, new low detection of MP at test of cure treated with extended azithromycin regimen and final resolution
7	17 June 2024	53	MSM	CT, NG, MG, TP	Dysuria and discharge	Azithromycin 1 g	Reinfection attributed to sexual partner failing to attend for testing and remaining untreated. Resolution after new treatment

MSM, men who have sex with men; MSW: men who have sex with women; MG, *Mycoplasma genitalium*; HSV, herpes simplex virus; NG, *Neisseria gonorrhoeae*; CT, *Chlamydia trachomatis*; TP, *Treponema pallidum*; MP, *Mycoplasma penetrans*.

Supplementary Table S1 lists pathogens related to urethritis detected in control patients, which included *C. trachomatis*, *N. gonorrhoeae*, *M. genitalium*, *U. urealyticum*, virus herpes simplex and adenovirus. Other STI-related microorganisms detected were HIV (*n* = 13), *T. pallidum* (*n* = 24, 4 acute cases and 20 past infections), and papillomavirus (*n* = 6). All of the pathogens related to urethritis were found in patients from group 2, and in 12 cases, more than 1 pathogen was detected simultaneously.

## Discussion

Bacterial species in the genus *Mycoplasma* are small wall-less bacteria that are very difficult to culture and grow slowly even on special media. Therefore, nucleic acid amplification tests are currently employed for their detection. As no commercial kits are yet available for detecting *M. penetrans*, for this study, an in-house RT-PCR assay was implemented, using primers described by Srinivasan et al. This approach has allowed *M. penetrans* to be described for the first time in Spain in samples from patients with urethritis.

In previous research, not focused on the potential association between *M. penetrans* infection and urethritis, this bacterial species was first detected in urine samples (Lo et al., 1991), and subsequently, has been found in samples from the urethra, pharynx, and rectum, especially in MSM living with HIV (Taylor-Robinson et al., 2003; Jian-Ru et al., 2012). In studies analysing larger numbers of samples, *M. penetrans* was detected in 24/1,541 (1.6%) urine samples from persons living with HIV in China (Chen et al., 2015), and in rectal samples of 31/231 (13.4%) men vs. 1/62 (1.6%) women with *C. trachomatis* infection in Denmark (Pérez-Prieto et al., 2022), the data supporting the association of *M. penetrans* with MSM sexual practices, as observed in earlier studies. In a recent study in the USA that analysed urethral microbiota using urine samples from MSM and MSW, *M. penetrans* was detected in 21/249 (8.4%) patients with urethritis vs. 2/182 (1.1%) without urethritis. Infection was associated with urethritis in MSM (17/129, 13.2% vs. 1/70, 1.4%) but not in MSW (4/120, 3.3% vs. 1/112, 0.9%) (Srinivasan et al., 2021). In contrast, in a study with urogenital samples from men with STI in France, *M. penetrans* was detected in 3.5% of MSM and 5.3% of HIV patients, but no association was found between infection and urogenital symptoms (Gardette et al., 2023). No studies have explicitly reported *M. penetrans* outside of human hosts, like other *Mycoplasma* species. Further research could provide important context for other possible environmental or non-sexual human transmission routes.

In our study, *M. penetrans* was detected in 7/55 (12.7%) of the cases of idiopathic urethritis, and in none of the controls with another cause of urethritis or who were asymptomatic, supporting the association of this microorganism with urethritis and consequently increasing the diagnostic yield. There is little knowledge about the transmission mechanisms of *M. penetrans*, although it is assumed to be sexually transmitted, based on available microbiological and epidemiological data. Transmission was observed across the 3 years of the study period without known relationships between the cases (probably community transmission), and it was more frequent in persons with a higher STI exposure (MSM with a history of STI). Although it was more frequently observed in MSM (16.2%) than in MSW (5.6%), the difference did not reach significance, likely due to the small sample size. Similarly, and as seen in other studies (Gardette et al., 2023), despite the fact that the positive cases were older than negative ones, which could be linked to behavioral patterns, the difference was not statistically significant. Previously, no differences in the transmission of this infection have been described based on origin or race, and in our study, five out of the seven *M. penetrans*-positive patients were Spanish born.

Six cases were successfully treated with azithromycin, and one resolved without antimicrobial treatment. There is currently little data available on the antimicrobial susceptibility of *M. penetrans*, which is intrinsically resistant to antimicrobials that act on the cell wall. Previous studies have stated that it is usually susceptible to macrolides, quinolones, and tetracyclines (Hayes et al., 1995), but strains with macrolide resistance have already been described (Duffy et al., 2000; Schwab et al., 2023). Resistance rates may increase, especially if the 1-g single-dose azithromycin regimen is used, and it may be necessary to administer extended regimes, as in one of our patients. Further, as has been happening with *M. genitalium*, as soon as molecular techniques become available for detecting 23S mutations associated with macrolide resistance, targeted antibiotic therapy should be used to optimize cure rates (Piñeiro et al., 2022).

A positive observation in this study was the notably small percentage of idiopathic urethritis (~5%), and this is attributable to the broad array of diagnostic techniques available and the STI clinic being located in the Microbiology Service, which minimises the delay to the start of diagnostic tests, optimising their yield. The main strength of this study is that two control groups were used, which has allowed us to associate *M. penetrans* infection with idiopathic urethritis, as it has not been detected either in the commensal microbiota in asymptomatic patients or as a coinfection in patients with pathogens causing urethritis. Nonetheless, the study has some limitations. It is a single-centre study, meaning our findings are not necessarily applicable to other study populations, even though the percentage of *M. penetrans*-positive cases detected suggests that this infection should investigated in other regions. Further, the small number of cases in the study impeded a more stratified analysis of the epidemiological characteristics associated with this infection.

## Conclusion

Our work contributes to the prevention, diagnosis, and treatment of urethritis, particularly in the context of underdiagnosed STIs. This case–control study provides evidence supporting the association between infection by *M. penetrans* and urethritis in men, especially MSM and men with a history of STIs, and notes resolution of symptoms following antibiotic treatment. To prevent the spread of this emerging microorganism, we suggest that its active searching should be performed in idiopathic urethritis cases. This is particularly important in populations at high risk of STI, to broaden the diagnostic and therapeutic landscape and improve control of these infections.

## Data Availability

The original contributions presented in the study are included in the article/Supplementary material, further inquiries can be directed to the corresponding author.

## References

[ref1] Chen L. S. Wu J. R. Wang B. Yang T. Yuan R. Zhao Y.. (2015). Epidemiology of Mycoplasma acquisition in male HIV-1 infected patients: a multistage cross-sectional survey in Jiangsu. China. Epidemiol. Infect. 143, 3327–3334. doi: 10.1017/S0950268815000461, PMID: 25792346 PMC9150968

[ref2] CNE. (2024). Vigilancia epidemiológica de las infecciones de transmisión sexual, 2022. Available online at: https://www.sanidad.gob.es/ciudadanos/enfLesiones/enfTransmisibles/sida/vigilancia/docs/Informe_Vigilancia_ITS_2022.pdf (Accessed March 14, 2025).

[ref3] Duffy L. B. Crabb D. Searcey K. Kempf M. C. (2000). Comparative potency of gemifloxacin, new quinolones, macrolides, tetracycline and clindamycin against Mycoplasma spp. J. Antimicrob. Chemother. 45, 29–33. doi: 10.1093/jac/45.suppl_3.29, PMID: 10824029

[ref4] ECDC (2024). “Chlamydia” in ECDC. Annual epidemiological report for 2022. Stockholm: ECDC.

[ref5] Gardette M. Touati A. Laurier-Nadalié C. Bébéar C. Pereyre S. (2023). Prevalence of *Mycoplasma penetrans* in urogenital samples from men screened for bacterial sexually transmitted infections. Open Forum Infect. Dis. 10:10. doi: 10.1093/ofid/ofad180, PMID: 37082616 PMC10111059

[ref6] Hayes M. M. Foo H. H. Timenetsky J. Lo S. C. (1995). In vitro antibiotic susceptibility testing of clinical isolates of *Mycoplasma penetrans* from patients with AIDS. Antimicrob. Agents Chemother. 39, 1386–1387. doi: 10.1128/AAC.39.6.1386, PMID: 7574538 PMC162749

[ref7] Jian-Ru W. Bei W. Hao C. Jin-Shui X. Xi-Ping H. (2012). Mycoplasmas in the urine of HIV-1 infected men. Epidemiol. Infect. 140, 1141–1146. doi: 10.1017/S095026881100104X, PMID: 21791147

[ref8] Lo S.-C. Hayes M. M. Wang R. Y.-H. Pierce P. F. Kotani H. Shih J. W.-K. (1991). Newly discovered mycoplasma isolated from patients infected with HIV. Lancet 338, 1415–1418. doi: 10.1016/0140-6736(91)92721-D, PMID: 1683419

[ref9] Pérez-Prieto I. Skafte-Holm A. Jensen J. S. (2022). High prevalence of *Mycoplasma penetrans* in *Chlamydia trachomatis* positive rectal samples from men: a brief report. Front. Microbiol. 13:914874. doi: 10.3389/fmicb.2022.914874, PMID: 35770176 PMC9234545

[ref10] Piñeiro L. Idigoras P. Arrastia M. Manzanal A. Ansa I. Cilla G. (2022). Increases in the macrolide resistance of Mycoplasma genitalium and the emergence of the A2058T mutation in the 23S rRNA gene: clonal spread? Antibiotics 11:1492. doi: 10.3390/antibiotics11111492, PMID: 36358147 PMC9686820

[ref11] Piñeiro L. Korta-Murua J. López-Cuesta S. Lasa I. Cilla G. (2019). Is the vertical transmission of *Chlamydia trachomatis* a little known problem in Spain? An. Pediatr 90, 395–397. doi: 10.1016/j.anpedi.2018.05.015, PMID: 29937303

[ref12] Rowley J. Vander Hoorn S. Korenromp E. Low N. Unemo M. Abu-Raddad L. J.. (2015). Chlamydia, gonorrhoea, trichomoniasis and syphilis: global prevalence and incidence estimates, 2016. Bull. World Health Organ. 97, 548–62P. doi: 10.2471/BLT.18.228486, PMID: 31384073 PMC6653813

[ref13] Schwab N. R. Young N. E. Nzenwata D. U. Toh E. Mikulin J. A. Wilson T. J.. (2023). Characterization of virulence-associated traits in *Mycoplasma penetrans* strains acting as likely etiological agents of idiopathic nongonococcal urethritis. J. Infect. Dis. 227, 1050–1058. doi: 10.1093/infdis/jiac505, PMID: 36588346 PMC10319971

[ref14] Srinivasan S. Chambers L. C. Tapia K. A. Hoffman N. G. Munch M. M. Morgan J. L.. (2021). Urethral microbiota in men: Association of Haemophilus influenzae and *Mycoplasma penetrans* with nongonococcal urethritis. Clin. Infect. Dis. 73, e1684–e1693. doi: 10.1093/cid/ciaa1123, PMID: 32750107 PMC8492123

[ref15] Taylor-Robinson D. Gilroy C. B. Keane F. E. (2003). Detection of several Mycoplasma species at various anatomical sites of homosexual men. Eur. J. Clin. Microbiol. Infect. Dis. 22, 291–293. doi: 10.1007/s10096-003-0910-x, PMID: 12734722

[ref16] Workowski K. A. Bachmann L. H. Chan P. A. Johnston C. M. Muzny C. A. Park I.. (2021). Sexually transmitted infections treatment guidelines, 2021. MMWR Recomm. Rep. 70, 1–187. doi: 10.15585/mmwr.rr7004a1, PMID: 34292926 PMC8344968

[ref17] Xu W. McDonough M. C. Erdman D. D. (2000). Species-specific identification of human adenoviruses by a multiplex PCR assay. J. Clin. Microbiol. 38, 4114–4120. doi: 10.1128/JCM.38.11.4114-4120.2000, PMID: 11060077 PMC87550

