# Supplementary material for: Mycoplasma penetrans urethritis in men. A case–control study
Source: Front Microbiol. 2025 Mar 19;16:1565685. doi: 10.3389/fmicb.2025.1565685 (PMC11961646; doi:10.3389/fmicb.2025.1565685)

Supplementary Figure 1. Bar chart figures comparing proportions of demographic characteristics between *Mycoplasma penetrans*-positive (N=7) vs. –negative (N=241) individuals: a) sexual behavior; b) age; c) origin.

a)

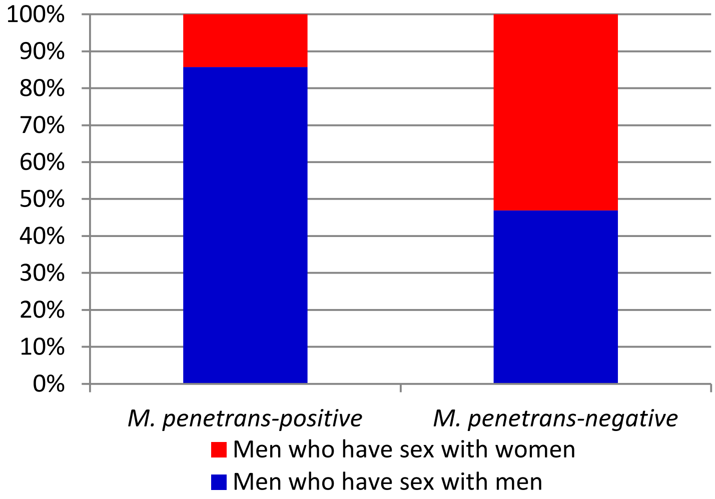

b)

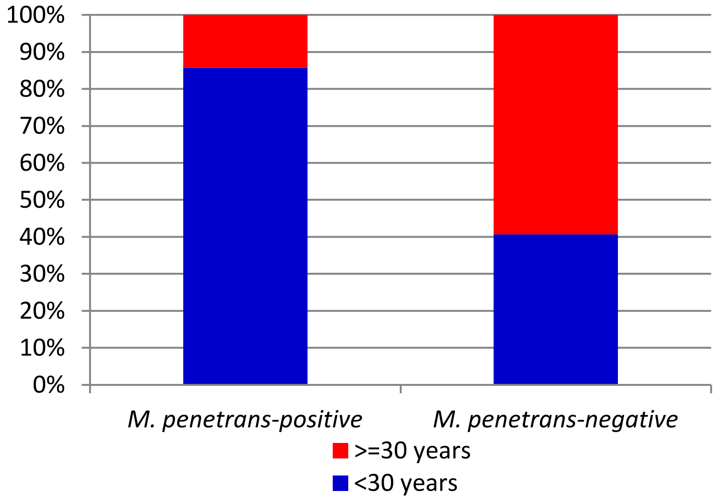

c)

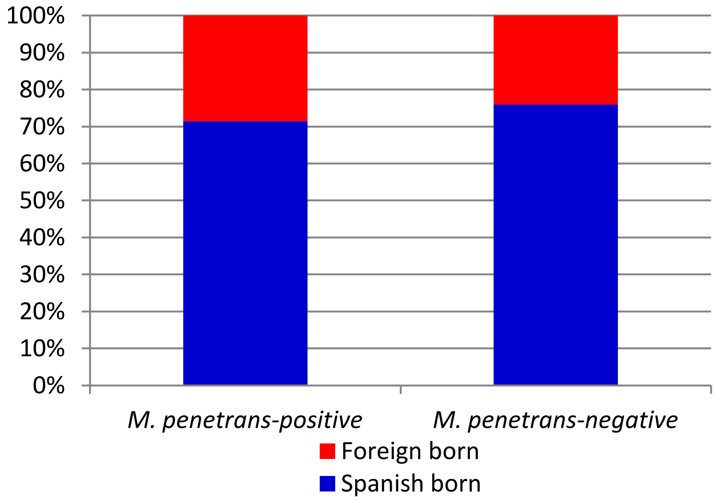

Supplement: Supplementary file 1 [file Data_Sheet_1.pdf]
