# Supplementary material for: Mycoplasma penetrans urethritis in men. A case–control study
Source: Front Microbiol. 2025 Mar 19;16:1565685. doi: 10.3389/fmicb.2025.1565685 (PMC11961646; doi:10.3389/fmicb.2025.1565685)
Supplement: Supplementary file 2 [file Data_Sheet_2.pdf]

Supplementary Table 1. Detailed epidemiological and clinical data of 248 men in which urethral samples were obtained to assess the association between *Mycoplasma penetrans* infection and urethritis (Donostia University Hospital, June 2021-2024); group 1 (N=55): cases of idiopathic urethritis; group 2 (N=102): controls of urethritis with a known pathogen; group 3 (N=91): asymptomatic controls.

**Case/control Group**	**Date**	**Age (years)**	**Origin[1]**	**Sexual behaviour**	**Previous STI, risk**	**Symptoms, signs, diagnosis**	**Gram stain**	**Micro-organism**	**Treatment**
G1C01	02/06/2021	30	Spain	MSM	MG	Dysuria, discharge	L2	MP	No
G1C02	19/01/2022	38	Europe	MSM	HSV	Dysuria, discharge	L2	MP	AZI-1
G1C03	11/09/2023	30	Spain	MSM	No	Dysuria, discharge	L3	MP	AZI-1
G1C04	19/12/2023	54	Spain	MSM	NG	Dysuria	L1,	MP	AZI-1
G1C05	27/05/2024	43	Spain	MSM	CT, NG, MG, TP	Inguinal adenopathy, mpox penis lesions	L1	MP	AZI-1
G1C06	30/05/2024	20	Africa	MSW	No	Dysuria, discharge	L2	MP	AZI-1
G1C07	17/06/2024	53	Spain	MSM	CT, NG, MG, TP, PrEP	Dysuria, discharge	L3	MP	AZI-1
G1C08	11/06/2021	30	Spain	MSM	No	Hematuria	L2	No	AZI-1
G1C09	07/07/2021	25	Spain	MSM	NG, MG	Dysuria, discharge	L2	No	AZI-1
G1C10	10/09/2021	44	Spain	MSM	HPV	Dysuria	L2	No	No
G1C11	02/10/2021	29	Spain	MSW	No	Dysuria, discharge, inguinal adenopathy	L2	No	AZI-1
G1C12	09/12/2021	32	America	MSM	HSV, PrEP	Dysuria, discharge, rectitis	L1	No	AZI-1
G1C13	17/06/2022	65	America	MSM	No	Dysuria, discharge	L-	No	AZI-1
G1C14	27/06/2022	48	Spain	MSW	NG, HPV	Orchiepididymitis	L1	No	DOXY
G1C15	13/07/2022	54	Spain	MSM	CT	Dysuria	L-	No	No
G1C16	19/07/2022	31	America	MSW	CT	Dysuria	L2	No	AZI-1
G1C17	12/08/2022	29	Europe	MSM	CT, HSV	Discharge	L2	No	AZI-1
G1C18	13/09/2022	41	Spain	MSM	MG, UU, TP	Dysuria, discharge	L3	No	No
G1C19	29/12/2022	28	Spain	MSW	CT	Asymptomatic, leukocyturia, USI, screening	L2	No	AZI-1
G1C20	03/01/2023	35	America	MSM	CT, NG	Dysuria, discharge	L2	No	AZI-1
G1C21	21/01/2023	42	Europe	MSM	NG	Dysuria, discharge	L2	No	AZI-1
G1C22	15/02/2023	18	Spain	MSM	CT, NG	Asymptomatic, leukocyturia	L2	No	No
G1C23	06/03/2023	22	Spain	MSW	No	Discharge	L-	No	No
G1C24	04/04/2023	32	Spain	MSW	No	Dysuria, discharge	L3	No	AZI-1
G1C25	04/05/2023	29	Spain	MSM	MG	Discharge	L-	No	AZI-1
G1C26	05/05/2023	47	Europe	MSW	No	Dysuria	L1	No	No
G1C27	08/05/2023	52	Spain	MSW	No	Hematospermia, dysuria	L-	No	No
G1C28	15/05/2023	27	Spain	MSM	No	Asymptomatic, USI, screening	L1	No	No
G1C29	15/05/2023	27	Spain	MSM	No	Discharge	L3	No	AZI-1
G1C30	22/05/2023	40	Spain	MSM	No	Asymptomatic, leukocyturia, USI, screening	L1	No	No
G1C31	20/06/2023	23	Spain	MSW	No	Dysuria, discharge	L3	No	AZI-1
G1C32	24/08/2023	47	America	MSM	CT, NG, TP	Dysuria	L1	No	No
G1C33	04/09/2023	24	Spain	MSW	No	Dysuria	L1	No	AZI-1
G1C34	07/09/2023	29	Africa	MSW	No	Dysuria, discharge	L1,	No	Unknown
G1C35	07/09/2023	30	Africa	MSW	No	Asymptomatic, leukocyturia, USI,	L1	No	Unknown

						screening			
G1C36	14/09/2023	28	Spain	MSM	No	Dysuria, discharge	L3	No	AZI-1
G1C37	21/09/2023	29	Spain	MSM	NG	Asymptomatic, leukocyturia, USI, screening	L1	No	No
G1C38	31/10/2023	43	Europe	MSM	NG, HIV	Dysuria, discharge	L3	No	No
G1C39	13/11/2023	31	Spain	MSW	NG	Dysuria	L2	No	AMOX
G1C40	15/12/2023	27	Spain	MSM	NG, MG	Discharge	L2	No	No
G1C41	18/12/2023	43	Europe	MSM	TP, HIV	Dysuria	L2	No	No
G1C42	28/12/2023	43	Europe	MSM	NG, HIV	Dysuria, discharge	L1	No	No
G1C43	29/12/2023	41	Spain	MSW	NG	Dysuria	L-	No	No
G1C44	12/01/2024	26	America	MSM	TP	Dysuria	L1	No	No
G1C45	18/01/2024	32	Spain	MSW	No	Asymptomatic, leukocyturia	L3	No	No
G1C46	26/01/2024	25	Spain	MSM	No	Dysuria, discharge	L2	No	No
G1C47	13/03/2024	31	Africa	MSM	No	Dysuria, discharge	L1	No	AZI-1
G1C48	08/04/2024	43	Spain	MSM	CT	Dysuria, discharge	L1	No	No
G1C49	15/04/2024	33	Spain	MSM	No	Asymptomatic, leukocyturia, sexual partner with diagnosed STI	L2	No	No
G1C50	11/05/2024	45	Spain	MSM	NG, MG, ADV	Discharge	L1	No	AZI-1, CEFT
G1C51	17/05/2024	33	Spain	MSM	NG	Dysuria, discharge	L2,	No	AZI-1
G1C52	30/05/2024	26	America	MSW	No	Asymptomatic, leukocyturia, USI, screening	L1	No	AZI-1
G1C53	14/06/2024	30	Spain	MSM	CT, MG	Dysuria, discharge	L1	No	DOXY, CEFT
G1C54	17/06/2024	37	Spain	MSM	NG, PrEP	Dysuria	L-	No	No
G1C55	17/06/2024	44	Spain	MSW	CT, NG, MG	Asymptomatic, leukocyturia, USI, screening	L2	No	AZI-1
G2c001	22/02/2022	46	Spain	MSW	No	Dysuria, discharge	L2,	CT	AZI-1
G2c002	05/07/2022	26	Spain	MSW	No	Discharge	L2	NG	CEFT
G2c003	11/07/2022	28	Africa	MSW	No	Dysuria	L2	CT	AZI-1
G2c004	19/07/2022	29	Spain	MSW	HINF	Dysuria, discharge	L3	CT	AZI-1
G2c005	19/07/2022	52	Spain	MSM	UU, TV	Dysuria	L2	UU	AZI-1
G2c006	09/08/2022	48	Spain	MSW	No	Dysuria	L2,	CT	AZI-1
G2c007	10/08/2022	27	Spain	MSW	CT	Dysuria	L2,	CT	AZI-1
G2c008	19/08/2022	34	Spain	MSM	No	Dysuria, discharge	L2	CT	AZI-1
G2c009	22/08/2022	38	Spain	MSM	No	Dysuria, discharge, conjunctivitis	L1	CT	AZI-1
G2c010	24/08/2022	21	Europe	MSM	HIV	Dysuria, discharge	L2	MG	AZI-1, MOXY
G2c011	08/09/2022	21	Africa	MSW	No	Orchiepididymitis	L-	CT	DOXY
G2c012	08/09/2022	23	Spain	MSW	No	Dysuria, discharge	L1	CT	AZI-1
G2c013	08/09/2022	32	Spain	MSM	CT	Dysuria, discharge	L3, GND	NG	CEFT
G2c014	08/09/2022	66	Spain	MSW	No	Dysuria, discharge	L3, GND	NG	CEFT
G2c015	22/09/2022	16	Spain	MSW	No	Dysuria, discharge	L-	UU, HSV	AZI-1, VALA
G2c016	23/09/2022	28	Spain	MSM	CT, NG	Genital lesions	L1	CT, TP	AZI-1, PEN-G
G2c017	30/09/2022	31	Spain	MSW	No	Dysuria	L1	CT	AZI-1
G2c018	03/10/2022	21	Spain	MSW	No	Dysuria	L1	MG	MOXY
G2c019	18/10/2022	32	Spain	MSM	CT, NG	Dysuria, discharge	L2	CT	AZI-1

G2c020	20/10/2022	20	Spain	MSW	No	Dysuria, discharge	L3, GND	NG	CEFT
G2c021	22/11/2022	20	Spain	MSM	No	Dysuria	L3, GND	NG	CEFT
G2c022	28/11/2022	43	Spain	MSW	No	Dysuria	L2	CT	AZI-1
G2c023	29/11/2022	40	Spain	MSW	No	Orchiepididymitis	L2,	CT	DOXY
G2c024	12/12/2022	33	Spain	MSW	MG	Dysuria	L2,	MG	AZI-X
G2c025	15/12/2022	30	America	MSM	TP	Discharge	L3,	CT	AZI-1
G2c026	27/12/2022	31	Spain	MSW	No	Dysuria, discharge, discharge	L3, GND	NG	CEFT
G2c027	04/01/2023	39	Spain	MSM	NG	Dysuria, discharge	L1,	MG	AZI-X
G2c028	04/01/2023	48	Spain	MSM	NG	Orchiepididymitis	L2	CT, NG	DOXY, CEFT
G2c029	19/01/2023	34	Africa	MSW	No	Dysuria, discharge	L3, GND	NG	CEFT
G2c030	23/01/2023	34	Spain	MSW	No	Dysuria	L3, GND	NG	CEFT
G2c031	24/01/2023	23	Spain	MSM	CT	Dysuria, discharge	L3, GND	NG	CEFT, DOXY
G2c032	30/01/2023	51	Spain	MSM	NG	Dysuria, discharge	L3, GND	NG	CEFT
G2c033	31/01/2023	43	Africa	MSW	UU	Dysuria	L-	GV	AMOX
G2c034	01/02/2023	21	Spain	MSW	No	Dysuria, discharge	L1	CT	AZI-1
G2c035	07/02/2023	37	Spain	MSW	No	Dysuria	L1	CT	AZI-1
G2c036	08/02/2023	45	Spain	MSW	No	Dysuria, discharge	L2	CT	AZI-1
G2c037	14/02/2023	23	Spain	MSM	NG	Dysuria, discharge	L3, GND	CT, NG	AZI-1, CEFT
G2c038	01/03/2023	38	America	MSW	No	Dysuria, discharge, orchiepididymitis	L1, EC2	CT	DOXY
G2c039	03/03/2023	35	Spain	MSM	CT, NG	Dysuria	L1, EC2	UU	AZI-1
G2c040	06/03/2023	40	Spain	MSM	No	Dysuria	L-, EC3	ADV	AZI-1
G2c041	13/03/2023	17	Spain	MSM	No	Dysuria, discharge	L3, GND	CT, NG	AZI-1, CEFT
G2c042	14/03/2023	36	Spain	MSM	No	Dysuria, orchiepididymitis	L2	CT	DOXY
G2c043	20/03/2023	21	Spain	MSW	CT	Dysuria, discharge	L3	CT	AZI-1
G2c044	24/03/2023	34	Spain	MSW	No	Dysuria, discharge	L2	MG	AZI-1
G2c045	28/03/2023	24	Spain	MSW	No	Dysuria, discharge	L3, GND	NG	CEFT
G2c046	28/03/2023	32	Spain	MSW	No	Dysuria, discharge	L-	NG	CEFT, AZI-1
G2c047	14/04/2023	32	America	MSW	No	Dysuria	L3	CT	AZI-1
G2c048	21/04/2023	27	Spain	MSW	No	Orchiepididymitis	L2	CT	DOXY
G2c049	24/04/2023	29	Spain	MSW	NG	Dysuria	L2	CT	Unknown
G2c050	24/04/2023	37	Spain	MSW	No	Dysuria	L1	NG	CEFT
G2c051	03/05/2023	25	America	MSW	No	Dysuria, discharge	L1	CT	AZI-1, CEFT
G2c052	04/05/2023	35	America	MSW	No	Dysuria	L1	CT	AZI-1
G2c053	09/05/2023	34	Spain	MSW	TP	Chancre, inguinal adenopathy	L2	MG	AZI-X, PEN-G
G2c054	11/05/2023	25	America	MSM	CT, NG, MG, HIV	Discharge	L3, GND	NG	CEFT
G2c055	12/05/2023	28	Africa	MSW	No	Dysuria, discharge	L2	ADV	AZI-1
G2c056	19/05/2023	29	Spain	MSW	No	Dysuria, discharge	L2	CT	AZI-1
G2c057	23/05/2023	32	Europe	MSM	No	Dysuria	L3, GND	NG	CEFT
G2c058	31/05/2023	29	Spain	MSW	No	Dysuria, discharge	L-	CT	AZI-1
G2c059	31/05/2023	19	Spain	MSM	No	Dysuria, discharge	L1	NG	CEFT
G2c060	31/05/2023	22	Spain	MSW	No	Dysuria, discharge	L-	HSV	VALA
G2c061	06/06/2023	41	Spain	MSW	No	Dysuria, discharge	L3, GND	NG	CEFT
G2c062	08/06/2023	29	Spain	MSW	CT	Dysuria, discharge	L-	CT	AZI-1
G2c063	09/06/2023	31	America	MSW	No	Dysuria	L1	CT	AZI-1
G2c064	12/06/2023	18	Spain	MSW	CT, NG	Orchitis	L-	CT	DOXY

G2c065	06/07/2023	36	Africa	MSW	No	Dysuria, discharge	L1	CT	AZI-1
G2c066	10/07/2023	49	Spain	MSM	CT, NG, MG	Dysuria	L3	CT	AZI-1
G2c067	10/07/2023	30	Spain	MSW	No	Dysuria, discharge	L1	CT	AZI-1
G2c068	14/07/2023	42	Spain	MSW	No	Dysuria	L2	CT	AZI-1
G2c069	14/07/2023	23	Spain	MSW	CT	Unknown	L1	CT	AZI-1
G2c070	27/07/2023	55	Spain	MSW	No	Dysuria	L3	CT	AZI-1
G2c071	28/07/2023	34	Spain	MSM	CT, MG	Dysuria, discharge, conjunctivitis	L3, GND	NG, ADV	CEFT
G2c072	11/08/2023	36	Asia	MSW	No	Dysuria, hemospermia	L2	CT	AZI-1
G2c073	11/08/2023	23	Spain	MSW	No	Discharge	L3, GND	NG	CEFT
G2c074	14/08/2023	41	Spain	MSM	HSV	Dysuria, discharge	L2, GND	NG	CEFT
G2c075	16/08/2023	21	Spain	MSW	No	Dysuria	L3	CT	AZI-1
G2c076	21/08/2023	27	Spain	MSW	CT	Dysuria, discharge	L1	CT	AZI-1
G2c077	23/08/2023	27	Spain	MSW	CT	Asymptomatic, USI, screening	L2	CT	AZI-1
G2c078	06/09/2023	48	Spain	MSW	No	Dysuria	L2	CT	AZI-1
G2c079	07/09/2023	34	Europe	MSW	No	Dysuria, discharge	L3, GND	NG	CEFT
G2c080	12/09/2023	19	Spain	MSM	No	Dysuria, discharge	L3, GND	NG	CEFT
G2c081	19/09/2023	19	Europe	MSM	No	Dysuria, discharge	L2	CT	AZI-1
G2c082	20/09/2023	41	America	MSM	NG, TP, HIV	Dysuria, discharge	L3, GND	NG	CEFT
G2c083	16/10/2023	30	Spain	MSM	No	Dysuria, discharge	L1	MG	AZI-X
G2c084	17/10/2023	24	Spain	MSM	NG	Dysuria, discharge	L3, GND	NG	CEFT
G2c085	20/10/2023	27	America	MSW	No	Dysuria	L1, GND	NG	CEFT
G2c086	06/11/2023	32	Spain	MSW	No	Dysuria, discharge	L3	UU	AZI-1
G2c087	28/11/2023	37	America	MSW	No	Discharge	L2	ADV	No
G2c088	15/12/2023	29	Spain	MSW	No	Dysuria, discharge	L2	CT, MG	AZI-1
G2c089	15/12/2023	19	Spain	MSM	No	Dysuria, discharge	L3, GND	NG	CEFT
G2c090	08/01/2024	52	Spain	MSW	CT, NG, MG	Dysuria	L3, GND	CT, NG	AZI-1, CEFT
G2c091	31/01/2024	35	Spain	MSW	No	Dysuria	L3	CT	AZI-1
G2c092	01/02/2024	30	Spain	MSW	NG	Dysuria	L2	CT	AZI-1
G2c093	06/02/2024	24	Spain	MSW	No	Dysuria	L1	CT	AZI-1
G2c094	16/02/2024	25	Spain	MSW	No	Dysuria, discharge	L2	CT	AZI-1
G2c095	20/02/2024	37	Spain	MSM	NG, UU	Dysuria, discharge	L3, GND	NG	CEFT
G2c096	10/04/2024	37	Spain	MSM	UU, PrEP	Dysuria	L-	No	No
G2c097	13/05/2024	29	America	MSW	NG	Discharge	L3	HSV-2	VALA
G2c098	17/05/2024	32	America	MSW	No	Dysuria, discharge	L3	CT	AZI-1
G2c099	23/05/2024	39	Africa	MSW	No	Dysuria	L1	CT	No
G2c100	23/05/2024	29	Europe	MSW	No	Orchiepididymitis	L2	CT	DOXY
G2c101	13/06/2024	28	Spain	MSW	UU	Dysuria, discharge	L-	UU	AZI-1
G2c102	28/06/2024	56	Spain	MSM	No	Dysuria, discharge	L-	UU	AZI-1
G3c001	20/03/2022	62	Spain	MSM	NG, TP, HIV	Asymptomatic, sexual partner with diagnosed STI	L-	No	No
G3c002	21/05/2022	40	Spain	MSM	CT, NG, HIV	Asymptomatic, sexual partner with diagnosed STI	L-	No	No
G3c003	08/07/2022	24	America	MSM	HPV	Asymptomatic, USI, screening	L-	No	No
G3c004	11/07/2022	22	Spain	MSW	No, recurrent balanitis	Asymptomatic, sexual partner with diagnosed STI	L-	No	No
G3c005	19/07/2022	41	Spain	MSW	No	Asymptomatic, previous STI, control	L-	No	No

G3c006	21/07/2022	33	Spain	MSM	TP	Asymptomatic, USI, screening	L-	No	No
G3c007	10/08/2022	30	Spain	MSM	No	Asymptomatic, sexual partner with diagnosed STI	L-	No	No
G3c008	11/08/2022	24	Spain	MSM	UU	Asymptomatic, USI, screening	L-	No	No
G3c009	17/08/2022	40	Spain	MSW	No	Unknown	L-	No	No
G3c010	19/08/2022	27	Europe	MSW	No	Asymptomatic, USI, screening	L-	No	No
G3c011	22/08/2022	50	Spain	MSW	TV	Asymptomatic, USI, screening	L-	No	No
G3c012	25/08/2022	39	Spain	MSM	CT	Asymptomatic, USI, screening	L-	No	No
G3c013	12/09/2022	26	Spain	MSM	CT	Asymptomatic, USI, screening	L-	No	No
G3c014	12/09/2022	31	Spain	MSW	CT, MG	Asymptomatic, USI, screening	L-	No	No
G3c015	22/09/2022	31	Spain	MSW	MG, UU	Asymptomatic, USI, screening	L-	No	No
G3c016	05/10/2022	24	Spain	MSM	MG, UU	Asymptomatic, USI, screening	L-	No	No
G3c017	06/10/2022	53	Spain	MSM	No	Asymptomatic, sexual partner with diagnosed STI	L-	No	No
G3c018	21/11/2022	24	Spain	MSM	No	Asymptomatic, sexual partner with diagnosed STI	L-	No	No
G3c019	22/11/2022	26	Spain	MSM	CT, MG	Asymptomatic, sexual partner with diagnosed STI	L-	No	No
G3c020	01/12/2022	44	Spain	MSM	CT	Asymptomatic, previous STI, control	L-	No	No
G3c021	01/12/2022	32	Spain	MSW	CT, MG	Asymptomatic, USI, screening	L-	No	No
G3c022	12/12/2022	33	America	MSM	MG	Asymptomatic, previous STI, control	L-	No	No
G3c023	12/12/2022	26	America	MSM	UU	Asymptomatic, USI, screening	L-	No	No
G3c024	26/12/2022	22	Spain	MSW	HSV	Asymptomatic, USI, screening	L-	No	No
G3c025	26/12/2022	35	Spain	MSW	No	Asymptomatic, USI, screening	L-	No	No
G3c026	03/01/2023	27	Europe	MSM	No	Asymptomatic, sexual partner with diagnosed STI	L-	No	AZI-1
G3c027	03/01/2023	33	Spain	MSW	No	Asymptomatic, USI, screening	L-	No	No
G3c028	19/01/2023	23	Spain	MSM	TP	Asymptomatic, USI, screening	L-	No	No
G3c029	23/01/2023	29	America	MSM	TP	Asymptomatic, USI, screening	L-	No	No
G3c030	30/01/2023	21	Spain	MSW	CT, TP	Asymptomatic, previous STI, control	L-	No	No
G3c031	31/01/2023	43	Spain	MSW	MG	Asymptomatic, USI, screening	L-	No	No
G3c032	10/02/2023	39	Spain	MSW	No	Asymptomatic, sexual partner with diagnosed STI	L-	No	No
G3c033	13/02/2023	26	Spain	MSM	CT, NG, MG	Asymptomatic, previous STI, control	L-	No	No
G3c034	27/02/2023	29	America	MSM	CT, NG, MG, TP	Asymptomatic, USI, screening	L-	No	No
G3c035	28/02/2023	29	Spain	MSM	NG	Asymptomatic, sexual partner with diagnosed STI	L-	No	No

G3c036	13/03/2023	37	Spain	MSM	CT, NG	Asymptomatic, USI, screening	L-	No	No
G3c037	14/03/2023	36	Spain	MSW	ADV, HPV	Asymptomatic, USI, screening	L-	No	CRYO
G3c038	20/03/2023	36	Spain	MSM	CT, NG, TP, HPV, mpox, HIV, HCV	Asymptomatic, sexual partner with diagnosed STI	L-	No	No
G3c039	21/03/2023	23	Spain	MSW	CT	Asymptomatic, USI, screening	L-	No	No
G3c040	27/03/2023	26	Spain	MSM	No	Asymptomatic, USI, screening	L-	No	No
G3c041	27/03/2023	45	Asia	MSW	CT	Asymptomatic, previous STI, control	L-	No	No
G3c042	18/04/2023	42	America	MSW	No	Asymptomatic, USI, screening	L-	No	No
G3c043	19/04/2023	33	America	MSM	MG	Asymptomatic, USI, screening	L-	No	No
G3c044	24/04/2023	31	America	MSM	CT	Asymptomatic, previous STI, control	L-	No	Unknown
G3c045	25/04/2023	37	Spain	MSM	TP	Asymptomatic, sexual partner with diagnosed STI	L-	No	No
G3c046	03/05/2023	25	Spain	MSW	CT	Asymptomatic, previous STI, control	L-	No	No
G3c047	10/05/2023	25	Spain	MSW	No	Asymptomatic, USI, screening	L-	No	No
G3c048	10/05/2023	41	Spain	MSW	No	Asymptomatic, sexual partner with diagnosed STI	L-	No	No
G3c049	22/05/2023	33	Spain	MSM	No	Asymptomatic, USI, screening	L-	No	No
G3c050	31/05/2023	32	America	MSM	NG, MG	Asymptomatic, USI, screening	L-	No	No
G3c051	05/06/2023	34	America	MSW	No	Asymptomatic, USI, screening	L-	No	No
G3c052	12/06/2023	27	Spain	MSM	NG	Asymptomatic, USI, screening	L-	No	No
G3c053	12/06/2023	43	Spain	MSW	CT, MG	Asymptomatic, sexual partner with diagnosed STI	L-	No	No
G3c054	07/07/2023	57	America	MSW	No	Asymptomatic, USI, screening	L-	No	No
G3c055	11/07/2023	24	America	MSW	No	Asymptomatic, USI, screening	L-	No	No
G3c056	12/07/2023	32	Spain	MSM	No	Asymptomatic, USI, screening	L-	No	No
G3c057	14/07/2023	45	Spain	MSW	NG	Asymptomatic, previous STI, TOC	L-	No	No
G3c058	14/07/2023	48	Spain	MSW	NG	Asymptomatic, previous STI, TOC	L-	No	No
G3c059	24/07/2023	28	Spain	MSM	CT, NG, TP	Asymptomatic, previous STI, control	L-	No	No
G3c060	26/07/2023	41	Spain	MSM	HIV	Asymptomatic, sexual partner with diagnosed STI	L-	No	No
G3c061	11/08/2023	37	Spain	MSW	CT	Asymptomatic, sexual partner with diagnosed STI	L-	No	No
G3c062	17/08/2023	25	Spain	MSM	MG	Asymptomatic, USI, screening	L-	No	No
G3c063	21/08/2023	50	Spain	MSM	HAV	Asymptomatic, sexual partner with diagnosed STI	L-	No	No

G3c064	23/08/2023	44	Spain	MSM	NG	Asymptomatic, USI, screening	L-	No	No
G3c065	31/08/2023	23	Spain	MSW	No	Asymptomatic, sexual partner with diagnosed STI	L-	No	No
G3c066	04/09/2023	29	Spain	MSW	CT	Asymptomatic, USI, screening	L-	No	No
G3c067	08/09/2023	30	Spain	MSM	CT, TP	Asymptomatic, sexual partner with diagnosed STI	L-	No	PEN-G
G3c068	08/09/2023	51	Spain	MSW	HPV	Asymptomatic, previous STI, control	L-	No	No
G3c069	22/09/2023	28	Spain	MSM	No	Asymptomatic, USI, screening	L-	No	No
G3c070	09/10/2023	19	Africa	MSW	No	Asymptomatic, sexual partner with diagnosed STI	L-	No	No
G3c071	16/10/2023	55	Spain	MSW	CT	Asymptomatic, previous STI, control	L-	No	No
G3c072	17/10/2023	39	Spain	MSM	NG, TP, HIV	Asymptomatic, USI, screening	L-	No	No
G3c073	23/10/2023	39	Spain	MSM	NG	Asymptomatic, USI, screening	L-	No	No
G3c074	24/10/2023	27	Spain	MSM	CT, NG	Asymptomatic, sexual partner with diagnosed STI	L-	No	No
G3c075	07/11/2023	41	Spain	MSM	NG, HIV	Asymptomatic, USI, screening	L-	No	No
G3c076	13/11/2023	43	Spain	MSM	NG, TP	Asymptomatic, USI, screening	L-	No	No
G3c077	11/12/2023	29	Spain	MSW	CT	Asymptomatic, previous STI, control	L-	No	No
G3c078	09/01/2024	44	Spain	MSW	No	Asymptomatic, USI, screening	L-	No	No
G3c079	02/02/2024	64	Spain	MSM	CT-LGV	Asymptomatic, previous STI, control	L-	No	No
G3c080	05/02/2024	45	Spain	MSW	No	Asymptomatic, USI, screening	L-	No	No
G3c081	07/02/2024	62	America	MSM	CT-LGV, TP, HIV	Asymptomatic, previous STI, control	L-	No	No
G3c082	08/02/2024	37	Spain	MSW	MG	Asymptomatic, USI, screening	L-	No	No
G3c083	21/02/2024	31	Spain	MSW	No	Asymptomatic, USI, screening	L-	No	No
G3c084	21/02/2024	49	Spain	MSW	No	Asymptomatic, USI, screening	L-	No	No
G3c085	26/02/2024	27	Spain	MSM	No	Asymptomatic, USI, screening	L-	No	No
G3c086	14/05/2024	35	Spain	MSW	CT, NG	Asymptomatic, previous STI, control	L-	No	No
G3c087	15/05/2024	45	Spain	MSM	CT, NG	Asymptomatic, previous STI, control	L-	No	No
G3c088	17/05/2024	29	Spain	MSM	CT, TP	Asymptomatic, USI, screening	L-	No	PEN-G
G3c089	27/05/2024	32	Spain	MSW	MG	Asymptomatic, previous STI, control	L-	No	No
G3c090	27/05/2024	23	Spain	MSW	No	Asymptomatic, sexual partner with diagnosed STI	L-	No	No
G3c091	17/06/2024	28	Spain	MSM	CT, NG	Asymptomatic, USI, screening	L-	No	AZI-1

(1): Europe, European countries other than Spain; G1: group 1; G2: group 2; G3: group 3; C/c(n): Case or control number; MSM: men who have sex with men; MSW: men who have sex with women; CT: *Chlamydia trachomatis*; LGV: lymphogranuloma

venereum; NG: *Neisseria gonorrhoeae*; MG: *Mycoplasma genitalium*; MP: *Mycoplasma penetrans*; UU: *Ureaplasma urealyticum*; HINF: *Haemophilus influenzae*; STRA: *Streptococcus agalactiae*; TP: *Treponema pallidum*; GV*: Gardnerella vaginalis*; TV: *Trichomonas vaginalis*; ADV: adenovirus; HAV: hepatitis A virus; HCV: hepatitis C virus; HIV: human immunodeficiency virus; HPV: human papillomavirus; HSV: herpes simplex virus; PrEP: pre-exposure prophylaxis; USI: unprotected sexual intercourse; STI: sexually transmitted infection; TOC: test of cure; HPF: high-power field; L-: <5 leukocytes/HPF and/or <20/ml leukocytes in urine; L1: 5-10 leukocytes/HPF or ≥20/ml leukocytes in urine; L2: 10-25 leukocytes/HPF; L3: >25 leukocytes/HPF; GND: gram-negative diplococci; AZI-1: azithromycin 1 g; AZI-X: extended azithromycin; DOXY: doxycycline; CEFT: ceftriaxone; PEN-G: penicillin G; AMOX: amoxicillin; MOXY: moxyfloxacin; VALA: valacyclovir; CRYO: cryotherapy.
